# Iron Metabolism at the Interface between Host and Pathogen: From Nutritional Immunity to Antibacterial Development

**DOI:** 10.3390/ijms21062145

**Published:** 2020-03-20

**Authors:** Marialaura Marchetti, Omar De Bei, Stefano Bettati, Barbara Campanini, Sandra Kovachka, Eleonora Gianquinto, Francesca Spyrakis, Luca Ronda

**Affiliations:** 1 Interdepartmental Center Biopharmanet-TEC, University of Parma, 43124 Parma, Italy; marialaura.marchetti@unipr.it (M.M.); stefano.bettati@unipr.it (S.B.); 2 Department of Food and Drug, University of Parma, 43124 Parma, Italy; omar.debei@studenti.unipr.it (O.D.B.); barbara.campanini@unipr.it (B.C.); 3 Department of Medicine and Surgery, University of Parma, 43126 Parma, Italy; 4 Institute of Biophysics, National Research Council, 56124 Pisa, Italy; 5 National Institute of Biostructures and Biosystems, 00136 Rome, Italy; 6 Department of Drug Science and Technology, University of Turin, 10125 Turin, Italy; sandra.kovachka@edu.unito.it (S.K.); eleonora.gianquinto@edu.unito.it (E.G.); francesca.spyrakis@unito.it (F.S.)

**Keywords:** nutritional immunity, siderophores, hemophores, *Staphylococcus aureus*, iron, virulence

## Abstract

Nutritional immunity is a form of innate immunity widespread in both vertebrates and invertebrates. The term refers to a rich repertoire of mechanisms set up by the host to inhibit bacterial proliferation by sequestering trace minerals (mainly iron, but also zinc and manganese). This strategy, selected by evolution, represents an effective front-line defense against pathogens and has thus inspired the exploitation of iron restriction in the development of innovative antimicrobials or enhancers of antimicrobial therapy. This review focuses on the mechanisms of nutritional immunity, the strategies adopted by opportunistic human pathogen *Staphylococcus aureus* to circumvent it, and the impact of deletion mutants on the fitness, infectivity, and persistence inside the host. This information finally converges in an overview of the current development of inhibitors targeting the different stages of iron uptake, an as-yet unexploited target in the field of antistaphylococcal drug discovery.

## 1. Iron and Nutritional Immunity

### 1.1. Iron: A Double-Edged Sword

Iron is very abundant in the biosphere, being the fourth most abundant element in the earth’s crust and the most abundant transition metal in the human body [1]. All living organisms, except for *Borrelia burgdorferi* [2] and some *Lactobacilli* [3], need iron to fulfill a plethora of biological functions. During evolution, iron has acquired a pivotal role in metabolism due to its favorable chemical properties that allow the formation of coordination bonds with electronegative atoms and the transition between the ferrous (Fe(II)) and ferric (Fe(III)) oxidation states. Indeed, iron can adopt different coordination states with different ligands containing oxygen, nitrogen, and sulfur. In hemoglobin (Hb) and myoglobin (Mb), for instance, iron is coordinated by the four porphyrin nitrogen atoms of protoporphyrin IX and a histidine residue known as proximal histidine [4]. Coordination can be incomplete, thus mediating a ligand transport function, as in the case of Hb, or a ligand-activation function, as in the case of oxygenases. In other cases, coordination can be complete, as in cytochromes, where iron shuttles between the two Fe(II) and Fe(III) redox states without changing its coordination environment [5]. The protein microenvironment also finely modulates the Fe(II)/Fe(III) redox potential that in most cases in living organisms is well below the standard value of +0.7 V [5,6]. In Hb, the heme iron is stabilized in the Fe(II) form within the hydrophobic heme pocket.

In aqueous solutions and in the presence of oxygen, iron is in the ferric oxidation state, which is poorly soluble [7,8], and needs to bind to proteins or hydrophilic chelators to be biologically available. Free iron is a double-edged sword, being essential for life but also extremely dangerous because of its chemical reactivity. Indeed, ferric iron participates in Fenton-type redox chemistry that generates reactive hydroxyl radicals, noxious for most macromolecules including proteins and DNA [9]. The search for a compromise between the versatility of iron as a protein cofactor and its potential adverse effects has shaped the evolution of systems, identified in plants, vertebrates, and invertebrates, that allow eukaryotes to store large amounts of this metal in a form unavailable to the bacterial invaders. Sequestration of iron within proteins has, thus, the double beneficial effect of limiting and modulating its reactivity and inhibiting bacterial proliferation. The concentration of free iron in human fluids was estimated to be around 10^−18^ M [10], several orders of magnitude lower than the concentration needed to sustain bacterial replication (low micromolar range, [11]). Iron withdrawal to limit nutrients available to bacteria and thus inhibiting their proliferation in the human host has been named “nutritional immunity” in the 1970s by Weinberg [12]. Nutritional immunity has a constitutive character, with basal expression of iron-binding proteins and the control on iron absorption to limit the concentration of free iron in the body. Moreover, this first-line defense is stimulated under infection/inflammation conditions and leads to the so-called hypoferremia of infection [13,14] (vide infra).

### 1.2. The Mechanisms of Nutritional Immunity

Nutritional immunity is a well-established first-line defense against pathogens and represents a very effective form of innate immunity that, indeed, allows invertebrates, like cockroaches [15], to survive in environments where bacteria proliferate [6]. The activation of innate immunity needs recognition by the human host of a specific pathogen-associated molecular patterns (PAMPs) by pattern recognition receptors (PRRs) [16,17]. An example of this mechanism of recognition of molecular determinants shared by multiple pathogens is mannose receptors, expressed on the surface of mammalian macrophages. Stimulation of PRRs leads to cytokine production and the induction of cytokines-dependent genes [17,18]. 

**Figure 1:**
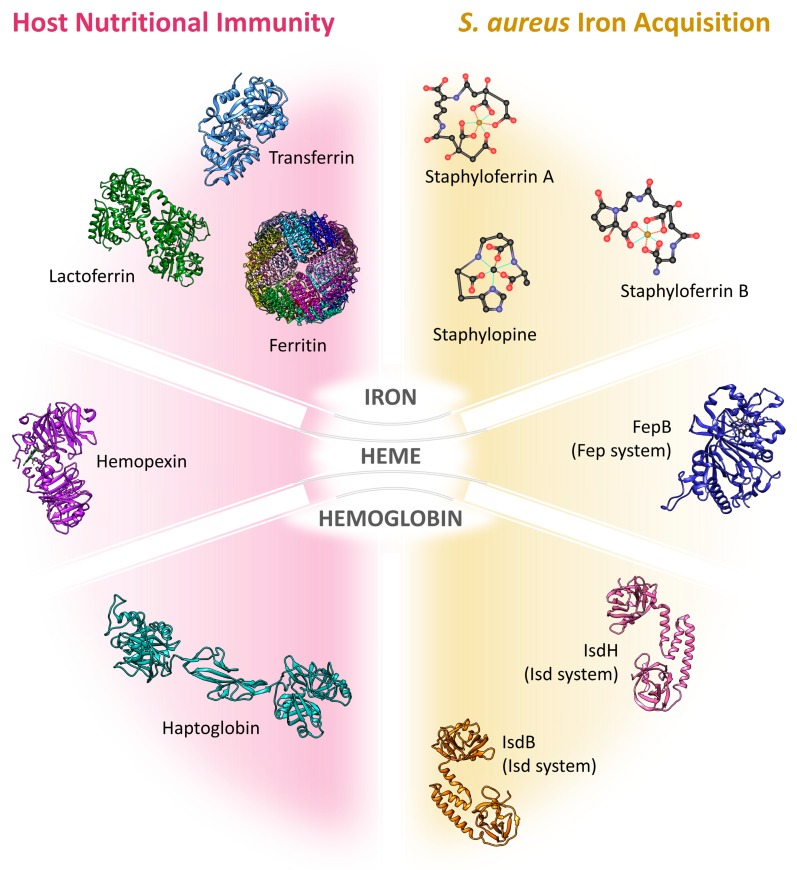
Schematic illustration of human proteins recruited in iron sequestration within the nutritional immunity (left), and *Staphylococcus aureus* effectors in iron retrieval (right). For protein representation, the following Protein Data Bank entries have been used: transferrin (1d3k), lactoferrin (1b0l), ferritin (1fha), hemopexin (1qhu), Hp 1-1 (extracted from 4wjg), IsdB^N1N2^ (extracted from 5vmm), IsdH^N2N3^ (extracted from 6tb2), FepB (3o72, illustration of the homolog *Escherichia coli* EfeB), and Hb/heme/iron (1a3n). Staphyloferrin A and B, and staphylopine have been modelled on the basis of Endicott et al. [46] and Deane [47] works, respectively. Proteins are divided into three groups based on the source of iron they exploit.

Iron absorption in the intestine is controlled by the expression of divalent metal transporter 1 (DMT1) on the luminal side of enterocytes [19,20] and, on the basal side, by ferroportin, that transports iron into the bloodstream. DMT1 actively transports iron into enterocytes and is also expressed on macrophages [18] and in endosomes during the transferrin cycle ([11] and references within). Transferrin cycle is a form of receptor-mediated endocytosis of iron-bound transferrin. The transferrin-receptor complex undergoes a pH-mediated conformational change in the endosomes with the release of free iron that is finally transported into the cytoplasm by DMT1. This proton co-transporter transfers only ferrous iron, thus Fe(III) must be reduced to Fe(II) by a specific reductase in the membrane before active transport takes place [11]. Iron is stored inside enterocytes and other cells like erythroblasts, hepatocytes, and macrophages in complex with ferritin (Figure 1). Ferritin is a ubiquitous protein playing a key role in iron detoxification and storage in a bioavailable form [21]. This heteropolymeric complex is formed by 24 subunits (L- and H-ferritin) and is able to bind up to 4500 iron ions behaving as a nanocage for this metal [22]. Ferrous iron is oxidized to ferric by the ferroxidase activity of the H-subunits and is stored inside the cage as crystallite with phosphate and hydroxide ions (ferrihydrite). Iron is transported throughout the body by serum transferrin (Figure 1), a protein of the transferrin superfamily that comprises serum transferrin itself, ovotransferrin and lactoferrin [23]. Ferroportin is the specific iron export protein that mediates iron efflux out of the cell in the ferrous form. This export protein allows iron absorption by enterocytes and iron efflux from macrophages [11]. Iron is immediately oxidized by a specific membrane ferroxidase and loaded as ferric iron onto transferrin for tissue distribution. Serum transferrin is the main iron transport protein in serum and has a typical bilobate shape, with a mixed α/β structure. The two lobes are connected by an unstructured linker region and undergo a large open-to-close transition upon iron binding [24,25]. Each transferrin molecule can bind two ferric iron ions, one in each lobe. The fraction of transferrin saturated by iron is about 30% in the healthy adult [10], which accounts for a free iron concentration of about 10^−18^ M. This saturation increases to 70% in the infant, due to a combination of moderate hyperferremia and a sub-optimal expression of transferrin. Under infection conditions, the concentration of free iron in body fluids is so low that transferrin saturation drops to 5%. Since transferrin is an acute-phase protein, the observed drop in transferrin saturation during infection results from a combination of increased plasma concentration of the protein and decreased iron absorption [6]. Iron bound to transferrin accounts for only 1% of total body content, whereas more than 80% of iron is sequestered inside heme-binding proteins, including Hb (60%), Mb (5%), cytochromes and iron-dependent enzymes. Lactoferrin (Figure 1) is secreted by exocrine glands and expressed and stored also in specific granules of neutrophils, from which it is released upon induction [26]. It was identified at concentrations as high as 8 mg/mL in many body fluids like colostrum, tears, sweat and seminal fluid [26]. Lactoferrin has a K_D_ for Fe(III) of 10^−20^ M [14,27], much lower than that of transferrin [8], supporting sequestration, rather than the transport function for the former protein [28]. Indeed, lactoferrin is able to uptake iron from transferrin and direct it to the liver. Transferrin receptors are expressed on macrophages, erythrocytes, hepatocytes, and virtually all cell types with few exceptions. Besides iron withdrawal, mammals have developed a series of mechanisms devoted to preventing iron overload upon degradation of heme-containing proteins. Since Hb binds the larger fraction of total body iron, most of the strategies for free-iron limitations concentrate on Hb/heme removal from circulation upon erythrocytes lysis. Haptoglobin (Hp) and hemopexin (Figure 1) have evolved to bind Hb and heme, respectively. Hp binds Hb with high affinity, with a K_D_ for dimeric Hb in the order of 10^−12^ M [14]. The circulating Hp reaches saturation at about 1.5 g/L free Hb [29] and is thus able to face efficiently moderate hemolysis. The Hp/Hb complex is specifically recognized by receptors on macrophages and hepatocytes [14,29] and readily degraded. Two polymorphic variants of Hp have been identified, Hp-1 and Hp-2, giving three different phenotypes: Hp1-1, Hp1-2, and Hp2-2, that differ in the quaternary structure and Hb binding capacity [30]. Hp2-2 is the phenotype with the lowest Hb binding capacity and is associated with a worse outcome of infections, higher cardiovascular risk and increased diet requirement of vitamin C due to the oxidative stress induced by free circulating heme [29,31]. Hemopexin is also an acute-phase protein expressed by hepatocytes that binds free heme with a K_D_ lower than 10^−12^ M and is recycled within hepatocytes once the complex is recognized by CD91 receptors [14]. Saturation of Hp and hemopexin by Hb and heme, respectively, has been reported to be more dangerous for the development of infections than transferrin saturation [10].

### 1.3. Nutritional Immunity under Infection Conditions

Under infection conditions, the basal nutritional immunity is reinforced, and additional mechanisms are activated to starve microbes of iron. The master regulator of iron uptake and distribution under infection conditions is the peptide hormone hepcidin. This 25- amino acid peptide is produced by the liver under physiological conditions and its expression is mainly regulated at the transcriptional level. The expression of the protein is positively regulated by hypoxia and active erythropoiesis and negatively regulated by a decrease in plasma iron concentration [32,33]. Under infection and inflammation conditions, interleukin-6 activates the expression of hepcidin through the Janus kinase/signal transducer and activator of transcription 3 (JAK/STAT3) pathway [8,33]. Stimulation of hepcidin expression leads to a decrease of iron efflux by a direct effect of the hormone on the amount of ferroportin expressed on cellular membranes of enterocytes and macrophages. Indeed, hepcidin binds to ferroportin and, after phosphorylation of a specific Tyr residue, directs the protein to degradation and thus controls iron efflux from cells [11]. Ferroportin is the only iron efflux pump in macrophages [8] and, in the enterocytes, mediates iron efflux through the basal membrane, thus regulating iron intestinal absorption. Owing to its role in iron cellular efflux, ferroportin degradation has two major consequences: (i) a decrease in iron absorption in the intestine and (ii) a decrease in the extracellular iron concentration following sequestration within macrophages. This innate immune response results in the iron starvation of extracellular pathogens and the increased availability of iron for intracellular pathogens, especially those residing in the macrophages like *Salmonella* ssp. However, macrophages also use iron to activate the oxidative burst that follows bacterial infection and the subtle interplay between iron availability to the pathogen and its effective use as antibacterial needs further clarification [8]. Also, lactoferrin is induced during infection and it is likely to support the iron-requiring reactions of the oxidative burst [26]. As mentioned above, also Hp and hemopexin expression is stimulated during the acute phase of bacterial infection, together with that of lipocalins. Lipocalins are a large protein family composed of structurally similar but functionally heterogeneous proteins that fulfil diverse functions [34]. Most of the family members show a distinctive β-barrel structure that folds in a cup-shaped cavity able to recognize and bind many different molecules ranging from retinol to dyes and pheromones. Specialized lipocalins, known as siderocalins, are able to bind bacterial siderophores [35], thus competing with bacterial mechanisms of iron uptake (see paragraph 2.1). Interestingly, bacteria have evolved the so-called “stealth” siderophores, non-canonical iron-chelating molecules that are not recognized by lipocalins. To date only *Salmonella* spp., *Klebsiella* spp. and *Bacillus anthracis*, but not *S. aureus*, have been reported to produce stealth siderophores [36,37]. Indeed, the number of siderophores produced by *S. aureus* is comparatively limited, but the bacterium is able to import exogenous siderophores including the stealth siderophore salmochelin (vide infra). Taken together, the above-mentioned mechanisms constitute the so-called hypoferremia/anemia of infection that leads to a decrease in the total iron concentration in body fluids from 10–30 micromolar to concentrations lower than 10 micromolar following infection by a pathogen [38]. The relevance of iron-withdrawal strategies in fighting bacterial infections is evinced by the reported increased susceptibility to bacterial, viral, and protozoan infections of individuals, whose ability to withdraw iron is compromised or who experience a sudden iron overload [39]. It is also historically supported by the ancient clinical practice of bloodletting, used for more than 2500 years to treat a variety of diseases, including bacterial infections [40]. Medical literature reports several examples of a positive correlation between plasma iron concentration and susceptibility to infections. However, in many cases the mechanism underlying increased susceptibility to infections is not completely understood. For these reasons, we report below some representative examples, and the reader is referred to the original works for a critical evaluation of the single cases. A good starting point is the review by Weinberg [39].

Newborns are especially susceptible to iron overload-related infections due to a less efficient transferrin production. One striking example is the seven-fold increase in septicemia and other bacterial infections in newborns treated with iron dextran in comparison with untreated neonates [10,39]. Iron overload can favor the shift from latent to manifest infections by, e.g., *Yersinia enterocolitica* and *Salmonella* [41,42]. Sickle-cell anemia and conditions displaying an increased rate of hemolysis are closely associated with an increased susceptibility to bacterial infections. For example, hemodialysis patients are more susceptible to infections-related morbidity and mortality [39]. The never-ending war between host and pathogens for iron acquisition is extraordinarily complex and especially fascinating in the case of commensal pathogens, like *S. aureus* and *Pseudomonas aeruginosa*, that can acquire the capability of infecting the host after a long period of cohabitation. Nutritional immunity can contribute in keeping the pathogen under control (asymptomatic colonization or localized disease) until some poorly characterized stimulus causes a shift to the infective phenotype. This review focuses on a commensal organism, *S. aureus*, that is listed among the most threatening pathogens in the World Health Organization priority list (a list of bacteria for which new antibiotics are urgently needed) [43]. *S. aureus* is also one of the most representative examples of the pathogens’ ability to acquire resistance to multiple, if not all, antibacterial drugs developed so far. This review has the intent of showing a potential way towards the exploitation of iron withdrawing strategies, selected by millions of years of evolution, in the treatment of staphylococcal infections [44,45].

## 2. Mechanisms of Iron Acquisition by *Staphylococcus aureus*

*S. aureus* acquires iron from several human host sources, depending on the site of infection and its growth phase [44,48,49,50,51,52]. During colonization and infection stages, *S. aureus* can settle in different niches and experiment alternative nutrient availability [53]. It is speculated that at the beginning of a bloodstream infection the bacterium can rely on the abundance of hemic iron bound to Hb in erythrocytes, which can be made accessible through the action of toxins such as hemolysins and leukocidins. In the scarcity of heme, e.g., in abscess environment, iron can be scavenged in inorganic form from the host storage proteins transferrin and lactoferrin by iron chelators known as siderophores. A consistent array of membrane and cell wall-anchored proteins are devoted to the detection of heme or iron sources [54,55]. It has been shown that *S. aureus* can extract heme also from Mb [56], but this process has been only limitedly investigated. This section is devoted to the presentation of the systems available to *S. aureus* to acquire iron from the host. The molecules involved in the mechanisms of iron acquisition are summarized in Figure 1 and the PDB codes to access the structural coordinates of the proteins involved are listed in Table 1.

### 2.1. Siderophores

Siderophores are low-molecular-weight compounds (500–1500 Da), widespread in plants, fungi, and bacteria, with high affinity for ferric iron and used to supply the cell under iron starvation conditions. In microorganisms, siderophores can acquire iron from host proteins like transferrin and lactoferrin, overcoming the nutritional immunity limitations. Their biosynthesis is regulated by iron levels and they are generally classified according to their iron-binding moiety: carboxylate, hydroxamate, catecholate, phenolate, and mixed-type. Like other pathogens, such as *P. aeruginosa*, *Salmonella* ssp., and *Escherichia coli*, *S. aureus* synthesizes and secretes siderophores in the extracellular space and, also, internalizes siderophores produced by other microorganisms. Siderophores, once bound to ferric iron, are imported inside the cell through an ATP-binding cassette (ABC) transporter, which is formed by an extracellular membrane-anchored soluble lipoprotein able to bind the substrate, a transmembrane permease, and an ATPase.

#### 2.1.1. Endogenous Siderophores

##### Carboxylate-Type Siderophores: Staphyloferrin A and Staphyloferrin B

**Figure 2:**
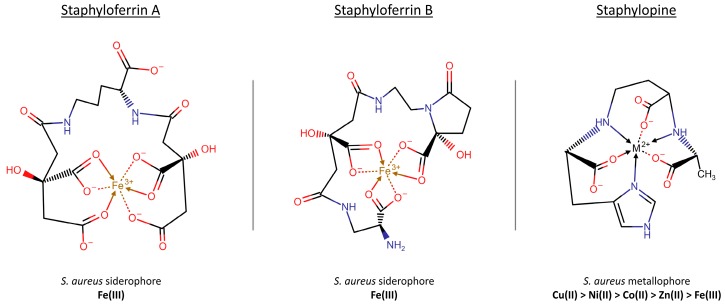
Structures of the three siderophores of *S. aureus,* adapted from Neumann et al. [106] on the basis of iron-bound models [46,47]. The chemical structures were drawn with Chemicalize.

*S. aureus* produces and secretes two main siderophores, staphyloferrin A and B (SA and SB, respectively), and expresses specific uptake systems. SA and SB both belong to the carboxylate-type siderophores (Figure 2). They are synthesized through a non-ribosomal peptide synthetase-independent siderophore (NIS) pathway, which is based on the alternation of dicarboxylic acids (such as succinate, citrate, and α-ketoglutarate) and diamines, amino alcohols and alcohols building blocks [84]. They are secreted by *S. aureus* during iron starvation and are primarily devoted to the scavenging of iron from extracellular precipitated ferric hydroxides and host proteins, like transferrin and lactoferrin, thanks to their higher affinity for Fe(III). SA and SB are reported to have different effects on infection outcomes in animal models and were recently shown by multimodal imaging mass spectrometry to have heterogeneous distribution across infection loci, suggesting specific roles rather than functional redundancy [50].

Staphyloferrins are transcribed from two different gene clusters, namely *sfa* and *sbn* for SA and SB respectively, both under Fur (ferric uptake regulator) repression. The synthesis of SA is carried out by two NIS synthetases, SfaD and SfaB, encoded by two genes divergently transcribed and acting sequentially by reacting two citrate molecules with D-ornithine [85]. SfaC racemizes L-ornithine to the D-enantiomer, providing the right substrate for the siderophore synthesis [85]. The citrate for SA biosynthesis is supplied by the citrate synthase CitZ of the tricarboxylic acid (TCA) cycle, hence linking the cellular energetic state to SA production [86,87].

Once synthesized, SA is exported from the bacterium by SfaA, homologous to transmembrane efflux pumps belonging to the major facilitator superfamily (MFS). Deletion of *sfaA* was shown to give a drastic reduction of staphyloferrin secretion in the medium, with a corresponding increase of its cytoplasmic levels in vitro [88], and to induce a growth defect in in vivo infection models [89]. When SA coordinates Fe(III), the uptake is managed by the Hts (heme transport system) ABC transporter [58], named for the role of heme uptake system originally attributed to this protein [53]. Hts complex is formed by the substrate-binding protein HtsA [57,58] and the integral permease heterodimer HtsBC. Since the operon (*htsABC*) is lacking a gene encoding for the ATP-binding protein, Hts activity relies on a promiscuous ATPase, FhuC [58]. HtsA binding to SA has a dissociation constant in the low/subnanomolar range [57].

The biosynthesis of SB is managed by the *sbn* gene locus (*sbnA–I*), which encodes all the enzymes needed for its synthesis (SbnABCEFGHI) [90]. This operon is found only in the most invasive coagulase-positive *S. aureus* strains, whereas the gene cluster for SA synthesis is widespread across both virulent coagulase-positive and commensal coagulase-negative strains [62]. SB is produced from one molecule of citrate and α-ketoglutarate and two molecules of L-2,3-diaminopropionic acid (L-DAP) and presents two isoforms, one with a linear and one with a cyclic hemiaminal α-ketoglutarate moiety. The latter form is likely the one physiologically present in solution [91]. It has been recently demonstrated that the citric acid stereocenter is important for the iron chelation properties of SB since it is necessary to create the coordination sphere around Fe(III) ion [91]. Differently from the biosynthesis of SA, the *sbn* gene cluster encodes also the enzymes for the synthesis of the three SB precursors (L-DAP), α-ketoglutarate (produced by SbnA and SbnB [92]), and citrate (SbnG). SbnA, a pyridoxal-5′-dependent enzyme, synthesizes N-(1-amino-1-carboxy-2-ethyl)-glutamic acid (ACEGA) and inorganic phosphate from *O*-phospho-L-serine (OPS) and L-glutamate [59]. SbnB then hydrolyzes oxidatively ACEGA in the presence of NAD^+^ to obtain L-DAP, α-ketoglutarate, and NADH [60]. Citrate is produced from oxaloacetate and acetyl-CoA by a structurally distinct type of citrate synthase, SbnG [61,93]. It has been observed that SbnG is not able to fully integrate the absence of CitZ for the production of SA in *citZ* mutant, thus posing the question of how citrate produced by SbnG is directed on SB biosynthesis [87]. *SbnI* encodes an L-serine kinase synthetizing OPS and, in addition, has a role in (i) the regulation of the staphyloferrin biosynthesis and (ii) the precursors biosynthesis [62]. In fact, it controls the expression of synthetase enzymes SbnC, SbnE, and SbnF and the decarboxylase SbnH [63], so controlling the siderophore export through the transporter SbnD. In addition, SbnI can bind a promoter sequence upstream of *sbnC*, controlling the transcription of part of the operon. Heme is a ligand of SbnI and inhibits SbnI binding to DNA, hence a mechanism has been proposed in which *S. aureus* senses intracellular heme and consequently modulates SB biosynthesis [94,95]. SB is exported outside *S. aureus* by SbnD to capture iron ions [94]. The fact that *sbnD* mutants have an only partial deficiency in SB secretion suggests that also other efflux mechanisms are available for this siderophore [88,89]. The following uptake of Fe(III)-SB is operated by the Sir (staphylococcal iron-regulated) system, which is encoded by the *sirABC* operon, adjacent to *snb* operon but transcribed in the opposite direction [96]. The ABC transporter is composed of the SirA lipoprotein (SB binding protein) [57], SirB and SirC transmembrane permeases and the promiscuous FhuC ATPase [97]. The dissociation constant between SirA and SB is in the low nanomolar range [57], similarly to the affinity of HstA for SA.

The utilization of Fe(III) bound to SA needs the activity of NtrA, a specific nitroreductase which releases iron from SA by its reduction of Fe(III) to Fe(II). On the other hand, the mechanism of dissociation of Fe(III) from SB inside the cytoplasm is still not characterized, but could be similar, involving a dedicated reductase. Alternatively, or in addition, unidentified iron chaperones could participate in iron transfer from SB to SA before the final release of the ligand [98]. However, since it has been observed that SA-deficient mutants are not showing a growth reduction when SB is still present, SA may not be the primary actor in the extraction of Fe(III) from transferrin, the main iron source in serum. Recently, it has been postulated that SA could promote colonization and infections of the skin and soft tissues, where glucose and iron concentrations are lower and higher, respectively, than in the serum. The comparison among the infection of a murine animal model with the *S. aureus* wild type and *sfa* and *sbn* deleted strains supports this thesis, demonstrating that an impaired SA synthesis affects the bacterial ability to form subcutaneous abscesses [99].

##### Other Endogenous Iron-Chelators

The existence of a third iron-specific siderophore, named aureochelin, was postulated by Courcol and colleagues [100]. Its structure, however, has never been characterized [101] and its significance in staphylococcal metabolism is still debated. In fact, the concentrated culture supernatant from a strain deleted in both staphyloferrins loci cannot support the wild type growth in iron-depleted conditions [58]. These observations suggest a prominent role of staphyloferrins in total siderophores production.

Together with other siderophores, *S. aureus* synthesizes a broad-spectrum metallophore known as staphylopine (StP). The machinery aimed at StP expression and trafficking is encoded by the *cntKLMABCDFE* operon [102,103,104] and its structure and function are similar to the phytosiderophores precursor nicotianamine, widely distributed in higher plants (Figure 2). StP biosynthesis is controlled by iron and zinc through the presence of *fur* and *zur* (zinc uptake regulator) boxes, and its activity is directed to the supply of copper, nickel, cobalt, zinc, and iron (in decreasing order of affinity) in the +2 oxidation state [102,103]. It has been demonstrated, both in vitro and in vivo, that an impaired efflux of StP leads to the metallophore accumulation in the cytoplasm, with detrimental effects on *S. aureus* growth [105].

#### 2.1.2. Exogenous Siderophores

When *S. aureus* is present in a bacterial community, it takes advantage of its ability to import and extract iron ions from siderophores produced by other bacteria (xenosiderophores). Xenosiderophores are imported thanks to the expression on *S. aureus* surface of specific uptake complexes highly conserved among different staphylococcal strains [54]. This competence permits the exploitation of molecules as ferrochrome, desferrioxamine B (DFO), aerobactin, coprogen, rhodotorulic acid, enterobactin, bacillibactin, salmochelin and 2,3-dihydrobenzoic acid [101,107,108,109]. These molecules can be grouped into two major categories, namely hydroxamate and catechol-type siderophores, based on their structures.

Hydroxamate-type siderophores bind Fe(III) and are imported by the Fhu import system, organized in an ATPase (FhuC), a heterodimeric permease (FhuBG), and two independently transcribed substrate-binding lipoproteins (FhuD1D2). FhuC acts as a promiscuous ATPase, serving several iron-uptake systems, as those involved in staphyloferrins import. FhuD1 and FhuD2 are homologous proteins whose genes reside outside of the *fhuCBG* cluster, evidence of evolutive genetic rearrangement of the staphylococcal chromosome [65,110]. Mutagenesis experiments highlighted a broader ligand binding ability for FhuD2 than FhuD1 [111].

Catechol-type siderophores’ efficacy in iron binding depends on the high affinity for Fe(III) and pH sensitivity of catechol. Moreover, they possess a very negative redox potential, giving a high metal selectivity for Fe(III) over Fe(II) [36]. *S. aureus* exploits bacterial catechol-based siderophores but also host catecholamine hormones [109,112]. The latter strip iron from transferrin upon reduction to Fe(II). Catecholamine-iron complex can be then internalized also in case the aerobic environment promotes iron reoxidation [112]. Both the categories can be internalized through the SstABCD ABC transporter [109,113]. The inactivation of *sst* locus impairs *S. aureus* viability in heart infection, posing questions about its role in endocarditis development [109].

FhuD1, FhuD2, and SstD, in comparison with other siderophore-binding proteins, have a lower affinity for their exogenous siderophore ligands (high nanomolar-low micromolar) but gain in broader binding capacity, a parasitic behavior which gives to *S. aureus* a growth advantage with respect to other bacteria [108,109,111].

Once imported inside the bacterial cell, *S. aureus* exploits the activity of the nicotinamide adenine dinucleotide phosphate (NADPH)-dependent iron uptake oxidoreductase (IruO) to extract iron as Fe(II) from hydroxamate siderophore and allows its metabolism [67,98]. NrtA is not required for extracting the iron bound to Fe(III)-DFO, while a transfer of ligand from DFO to SA is possible through the action of IruO, which dissociates iron from DFO; iron is then chelated by apoSA and released again by NrtA [67,98]. This is a unique example of ligand transfer between siderophores, that could involve intermediate protein carriers not yet discovered. Interestingly, *iruO*-deleted strain USA300 does not increase its virulence during DFO treatment, an opposite phenotype than the isogenic wild type strain that usually threatens patients’ life assuming DFO concomitantly to a staphylococcal infection [98]. 

The extraction of iron from catechol-type siderophores in bacteria generally involves the destruction of the carrier through the action of an esterase, with the concomitant release of unreduced Fe(III) [114,115,116]. *S. aureus* could likely use a similar mechanism, but a homologous esterase has not yet been identified.

### 2.2. Hemic Iron

#### 2.2.1. Hemolysins and Leukocidins

The primary supply of iron for *S. aureus* seems to be heme [53], also known to be the most available and abundant iron source in the mammalian host (see Section 1.2). Heme is bound to globin proteins, mainly Mb in the muscles and Hb in the blood. Since Hb is strictly compartmentalized, *S. aureus* releases an arsenal of toxins able to kill immune system cells, overcome epithelial and endothelial tissues, and lyse erythrocytes to provide iron for bacterial growth. 

The pore-forming cytotoxins can be divided into three main groups, namely Hla, Hlb, and Hld, based on their structure and mechanism of action. Their production is controlled by the regulatory proteins Sae (*S. aureus* exoprotein expression system), Agr (accessory gene regulator) and Sar (staphylococcal accessory regulator) to respond to changing microenvironments [117,118,119]. The first group is represented by α-hemolysin (Hla), the more extensively characterized hemolysin, classified as a very relevant virulence factor [68]. Hla is a homoheptameric transmembrane β-barrel forming an aqueous pore that allows the diffusion of low molecular weight molecules (cut-off 1–4 KDa) such as ions and ATP [120], leading to necrotic death of the target cell. This toxin is important for *S. aureus* virulence in pneumonia, sepsis, septic arthritis, brain abscess and corneal infections [121,122,123,124]. As is the case with Hla, γ-hemolysin (Hlg) and LukED leukocidin are also barrel-forming proteins but assemble as hetero-oligomers and are known as bicomponent toxins. Their action is central in the lysis of human erythrocytes [125]. Hlg is a potent toxin functional as HlgA/HlgB or HlgC/HlgB pairs [69], forming hetero-octameric pores on erythrocytes (HlgAB) and neutrophils (HlgCB) membrane and able to enhance the survival of *S. aureus* in human blood [126,127]. LukED [70] is one of the most important *S. aureus* virulence factors and plays a critical function in pathogenesis. In fact, the deletion of *lukED* in a highly virulent strain results in a remarkable attenuation in mouse model [128,129]. Recently, experimental data demonstrated the lytic capacity of LukED on erythrocytes and the inhibition of this process by LukSF-PV (Panton–Valentine leukocidin) [125,130]. 

The second group of pore-forming cytotoxins comprises the enzyme sphingomyelinase C (β-hemolysin, or Hlb) but, since its gene is frequently interrupted by the insertion of mobile genetic elements, its role in virulence is considered to be limited [131].

The third group is represented by membrane-damaging peptides as δ-hemolysin (Hld, identified as one of the phenol-soluble modulins, PSMs [132]), an amphipathic peptide with antimicrobial activity and involved in the promotion of allergic response by the host during skin colonization [72,133,134].

Besides their lytic activity, these proteins mediate several moonlighting functions as receptors activation, interference with immune response pathways, adhesion and biofilm regulation, antimicrobial activity [135]. Very importantly, the secretion of Hla, Hld, and Hlb, in synergy with other factors, participates in *S. aureus* internalization, persistence, and escape from the phagosome in non-specialized phagocytic cells [136,137,138] endowing the bacterium with an alternative dissemination pathway.

The *hlb*, *hld,* and *hlg* loci coding for these toxins are present in the majority of *S. aureus* strains, with *hla* almost always present, whereas the conservation of *luk* locus is still debated [135,139].

#### 2.2.2. Isd System

##### Isd in Heme Uptake

The iron-regulated surface determinant (Isd) system is composed of nine Isd proteins acting sequentially, from heme uptake from Hb and Hp-Hb complex, to internalization and iron release [55]. The Isd proteins are encoded within five operons (*isdA*, *isdB*, *isdCDEFsrtBisdG*, *isdH*, and *orfXisdI*), all presenting an upstream *fur* box controlling their transcription. The first steps of heme acquisition take place in the extracellular environment, requiring the action of several peptidoglycan-anchored proteins. The exposure of this array of receptors is the charge of two sortase transpeptidases, SrtA [73] and SrtB [74,75], recognizing different signal patterns on target proteins (LPxTG and NPQTN, respectively [140,141,142]. The gene *srtB* is inside the Isd cluster, while *srtA* sequence is outside of this genomic region; the transcription of both proteins is controlled by Fur. Mutants carrying the deletion of A or B sortases loci are characterized by a reduced virulence and colonization ability (Table 2) [142]. 

IsdA, IsdB, IsdC, and IsdH (previously called HarA [143]) are bound to the peptidoglycan surface, with a different degree of penetration [55]. IsdB and IsdH are the more extracellularly exposed, IsdA is only partially exposed, while IsdC is almost totally inserted in the peptidoglycan layer. These four proteins have a C-terminal signal for sortases; SrtA anchors IsdA, IsdB, and IsdH [55,143,144,145], while SrtB anchors IsdC [142]. They all share the same modular structure, composed by domains called NEAT (NEAr Transporter) [146], able to bind heme, Hb or Hp-Hb complexes. 

IsdB and IsdH perform the first step in hemic iron acquisition since they are able to bind Hb [76,77] or the Hp-Hb complex [78] and extract the heme, two steps operated by distinct NEAT domains. It has been observed that, while the two Hb receptors have the same heme acquisition capability, only IsdB seems to be the determinant necessary to *S. aureus* for virulence and proliferation in mouse model [56,147,148,149,150,151]. Remarkably, Pishchany and coworkers emphasized a preferential IsdB binding to human Hb (hHb) rather than mouse Hb (mHb), with a difference of an order of magnitude in the affinity of the complexes in vitro (5.5 × 10^−8^ M for hHb versus 9.8 × 10^−7^ M for mHb) [148]. This characteristic is conserved among the clinically relevant *S. aureus* strains, warning about possible misleading results of pathogenesis studies carried out in mouse models. For this reason, the authors introduced the use of a mouse model carrying hemizygous humanized Hb [147,148].

Once captured by IsdB or IsdH, heme is unidirectionally transferred to IsdA and then to IsdC through a NEAT-to-NEAT transfer [79,80,152,153]. IsdA and IsdC, besides their hand-pass role with extracted heme from upstream to downstream Isd proteins, can also perform a self-transfer reaction based on self-dimerization [154]. The heme bound to IsdC can be translocated across the cell wall and transferred to IsdE, an ABC transporter-binding lipoprotein [81,154]. The role of IsdC is to facilitate the transfer of the heme from IsdA to IsdE acting as the “central cogwheel” [155]. Heme is then transported inside the cytoplasm thanks to the IsdDEF ABC transporter [55]. The heme inside the cytoplasm can be degraded by IsdG [82] or IsdI [83], both presenting a heme oxygenase activity [156]. These proteins are able to distort the heme (heme ruffling), facilitating the formation of the oxidation products 5-oxo-δ-bilirubin and 15-oxo-β-bilirubin—also called staphylobilins—and formaldehyde [157,158].

IsdI and IsdG are paralogous enzymes, probably acting selectively in the different microenvironments encountered by *S. aureus*. This hypothesis is sustained by in vivo studies, where strains deleted in *isdG* or *isdI* genes show different levels of virulence with respect to wild type in different organs during infection in mice [159]. Since IsdI is able to transfer heme to SbnI causing its inhibition, it could have a regulatory function in SB synthesis [160], thus linking heme and inorganic iron acquisition (see Section 2.1.1).

The activity of IsdI and IsdG is also supported by other two heme degrading proteins, IruO oxidoreductase and NtrA nitroreductase, active in extracting iron from SA and hydroxamate siderophores. IruO and NtrA can degrade heme and facilitate iron release from the porphyrinic ring [98,161].

##### Isd Moonlighting Activities

Proteins participating in heme acquisition through the Isd system possess other important moonlighting functions, involved in immune evasion and adhesion [162]. In iron starvation conditions, for example, IsdA is expressed on *S. aureus* surface and, through the polar nature of its C-terminal domain, confers resistance to the antimicrobial action of serum fatty acids present on host skin as an innate defense. Patients affected by atopic dermatitis, possessing consequently lower levels of fatty acids, are more susceptible to skin colonization [163]. Moreover, IsdA NEAT domain inhibits apo-lactoferrin proteolytic activity and binds human transferrin [164]. IsdH has been proposed to promote immune evasion by interfering with complement proteins and opsonophagocytosis process during systemic infections [165]. Several Isd proteins, on the other hand, play the additional role of adhesins, thanks to their modular structure composed of NEAT domains. They mediate the adhesion to host tissues and synthetic surfaces coated with plasma proteins, such as fibronectin, fibrinogen, and vitronectin [162,166]. This is the case of IsdC [167], IsdA [145,164], and IsdB [168,169].

#### 2.2.3. Fep System

The Fe-dependent peroxidase (Fep) system has been identified in *S. aureus* by Biswas and coworkers in 2009 and its function has been implicated in the extracellular iron extraction from heme while preserving the tetrapyrrole ring intact [170,171]. The system, whose expression is regulated by Fur, comprises three proteins: FepA, predicted to be a membrane-anchored lipoprotein; FepB, an iron-dependent peroxidase with a typical twin-arginine translocation (TAT) signal peptide that enables its exportation by the TAT translocon; FepC, an integral membrane protein. FepABC system is homologous to EfeUOB system from *E. coli*, which has been shown to recognize heme and allow periplasmic hemic iron extraction through a deferrochelation reaction [172]. It has been proposed that FepB can bind heme and reduce iron, then the ferrous ion is internalized by the bacterium through the action of the membrane-associated proteins FepA/FepC, whose specific role is still unclear [171]. In addition, FepA and FepB are possible interactors, as their respective functions cannot be complemented by the homologous *E. coli* partners [171]. The physiological relevance of this process has not yet been established, although it could be implicated in external iron detoxification during oxidative bursts [170]. 

Fep-TAT system is not homogeneously conserved among staphylococcal subspecies but, with few exceptions, is generally found in the most aggressive human strains as *S. aureus*, *Staphylococcus haemolyticus,* and *Staphylococcus lugdunensis*. Moreover, in vivo experiments demonstrated that *S. aureus* RN1HG *ΔtatAC* and *Δtat-fep* mutants show an average 1-log decreased bacterial load in comparison with the wild type in a mouse hematogenic kidney abscess model [170].

### 2.3. Heme and Iron Homeostasis inside S. aureus

Once internalized, iron and heme follow specific fates, balancing the nutritional needs with storage and preservation of the bacterium from potential toxicity. Hemic iron can be released by IsdG or IsdI through their oxygenase activity or, based on the “heme hijacking hypothesis’’, the heme acquired from the host can be exploited as a cofactor in the bacterial heme-proteins, for example in cytochromes [53,173,174]. This process could possibly be energetically favorable to *S. aureus*, explaining its preference for heme as an iron source. Heme homeostasis inside the cell is maintained through the action of the efflux pump HrtAB (heme regulated transporter), which pumps the excess of heme outside the bacterium. The transcription of *hrtAB* is activated by the dimeric heme sensory system HssRS, which is sensitive to the intracellular concentration of heme [175,176,177]. 

*S. aureus*, like other microorganisms (e.g., *E. coli*, *Helicobacter pylori*, *Porphyromonas gingivalis,* and *Campylobacter jejuni*), can store the excess of inorganic ferric iron inside the ferritin-like protein FtnA [178]. The function of bacterial ferritins differs significantly among bacterial species, from iron source, to sustain maximal growth in iron starvation, to protection against Fenton chemistry during oxidative bursts. The staphylococcal ferritin gene *ftnA* encodes for 19.5 kDa subunits that, based on the high homology with previously identified ferritins, assemble in a 24-mer protein able to oxidize Fe(II) to Fe(III) [179,180]. FtnA shares an elevated degree of identity also with eukaryotic ferritins, conserving the residues involved in iron binding and ferroxidase center formation [181]. In *S. aureus*, the transcription of *ftnA* is controlled by PerR (peroxide-responsive repressor), based on nutrient availability for *S. aureus* growth [178,181]. PerR is also implicated in the expression of enzymes responsible for hydroxyperoxides scavenging and this parallel regulation can be attributed to the necessity to lock reactive iron during oxidative bursts [182]. This hypothesis is further supported by the PerR-dependent regulation of the Dps homolog MrgA (metallo-regulated gene A), a protein with iron-chelating function that protects DNA from oxidative stress damage [178].

## 3. Effects of Iron Restriction and Mutations of Assimilatory Pathways on Fitness and Virulence

In this section, a survey of the major consequences on *S. aureus* virulence and fitness of limiting iron acquisition are reported. A more detailed list of deletion mutants and their effect on virulence in animal models of infection is given in Table 2, together with relevant references. 

Iron availability is central in bacterial persistence and drives, among the others, the genic regulation of several operons involved in its acquisition, metabolism, and storage. *S. aureus* mainly controls iron uptake and oxidative stress response through Fur, a highly conserved transcription modulator that binds an inverted repeat target sequence (Fur box) and is present in many Gram-negative and low-GC-content Gram-positive bacteria. In *S. aureus*, it is estimated that the Fur regulon comprises nearly 20 transcription units involving around 50 genes, directly or indirectly controlled. The simplest regulation mechanism proposed for Fur involves the regulator to be bound to DNA as a Fe(II)-Fur dimer in iron repletion condition, whilst iron depletion promotes the dissociation of monomeric apo-Fur from the target sequences, allowing DNA transcription. Concomitantly, many studies also reveal the existence of several genes requiring Fur as expression activator [190,191,192], highlighting a complex and multifactorial influence of this protein on bacterial metabolism. Importantly, Fur participates in the expression regulation of virulence and immunomodulatory factors and genes involved in oxidative stress defense (in particular the catalase-coding gene *katA*), a metabolism intimately connected to the presence of iron inside the bacterial cell. This role is played together with the transcription regulator PerR, in turn, responsible for the regulation of Fur, alkyl hydroxyperoxide reductase AhpC and bacterial ferritins FtnA and MrgA expression [178,181,191]. As a consequence, mutant strains lacking *fur* show a decreased fitness during infection and are more susceptible to neutrophil-mediated killing [193], most likely due to an impairment in oxidative stress defense and the expression of virulence factors. Interestingly, it was demonstrated that the inactivation of Fur in *E. coli* leads to an increased antibiotic resistance insurgence, related to iron-overload-dependent mutagenesis [194]. Indeed, *fur* deletion mimics an iron-depleted condition inside the cell and, on one hand, promotes the transcription of the bacterial iron transporters and, on the other, avoids the production of iron-storage proteins, perturbing iron homeostasis and inducing oxidative stress. Almost all the operons involved in iron uptake rely on a direct Fur regulation, ruled by the presence of upstream Fur boxes [90,142,170,191,195,196,197]. However, as previously described, *S. aureus* possesses redundant systems for iron supply and it was demonstrated that the preferential iron source during early infection is the heme from host hemoproteins [53], the latter being the most abundant source since the inorganic form is strictly sequestered. Heme was found to be a direct regulator of SB biosynthesis [95] and the activator of HssSR system for heme detoxification. Interestingly, deletion of *hssSR/hrtAB* genes confers a more virulent phenotype to *S. aureus* in liver infection, with effects beyond the regulation of heme homeostasis [175,176,177]. The lack of these genes, in fact, alters the expression and secretion of several immunomodulatory proteins that impair the recruitment of phagocytes and granulocytes, challenging host survival. These observations disclose a complex regulation of iron sensing, where the iron uptake activation depends on nutrients availability in the specific environment encountered in the infection district and Fur plays a prominent role in virulence establishment [192,193]. In fact, Fur action overlaps with that of other important regulation factors, such as Agr, Sae, and Rot (repressor of toxins) [192], creating a complex and still not fully understood signaling network. Together, based on iron availability, these proteins control bacterial physiology and virulence through the expression of hemolysins, adhesins, leukocidins, and drive the formation of biofilms. For instance, Hla, HlgC, and LukED were found to be upregulated in a *fur*-deleted strain [193]. This complex interplay is mostly indirect and likely mediated also by the transcription of small RNAs, whose metabolism is a matter of several studies. Recently, the sRNA S596 (homologous to *E. coli* RyhB and controlled by Fur [198]) was identified and predicted to repress the expression of iron-related genes and TCA cycle enzymes like the citrate synthase CitZ, controlling SA production [199,200]. This metabolic rearrangement tuned by Fur is known as “iron-sparing response” and decreases the iron need of the bacterium through a limitation in non-essential iron-containing enzymes expression. The TCA cycle, that comprises iron-sulfur clusters-depending enzymes, is one example of this metabolic switch. In fact, *S. aureus* can upregulate the glycolytic and fermentative pathways, compensating its energetic requirements [87]. In this situation, *S. aureus* multifaceted ability to acquire iron allows the SB biosynthesis even in the absence of TCA citrate, sustained through SbnG-I catalytic activities (see Section 2.1.1) [62].

Staphylococcal fitness in host invasion is partly attributed to the proficiency in iron acquisition through multiple mechanisms. Each of them relies on protein complexes, employing at least one ABC-transporter importing iron across the membrane, an ATPase and a membrane-anchored lipoprotein [54,201]. This last type of proteins belongs to the class known as iron-regulated lipoproteins (IRLPs) and utilizes the acylated moiety in the recognition and binding of iron, driving its internalization across the membrane through the cognate transporter. IRLPs are recognized as PAMPs by the Toll-like receptor 2 (TLR2), influencing the host innate immune response [202,203,204]. It has been demonstrated that IRLPs, despite inducing an immune response, enhance bacterial fitness and are fundamental in persistence of infection [205]. Thus, mutant strains lacking the enzyme lipoprotein diacylglyceryl transferase (Lgt, responsible for IRLPs acylation) evoke the expression of a different array of cytokines than wild type *S. aureus*, weakening the host antimicrobial response, and, more importantly, are unable to adapt their iron acquisition strategy in response to the inflammation-dependent restriction [205]. In addition, the length of the acyl group influences the recognition ability of the immune system, balancing the presence of *S. aureus* as a commensal or a pathogen [204].

## 4. Antibiotic Strategies Targeting Iron Uptake in *S. aureus* - Small Molecules

In the present section, we review the iron-related systems that can be targeted, or exploited, for the development of possible antimicrobials and the strategies investigated up to now. The section has been divided into paragraphs that report targets belonging to pre-iron-uptake systems (secretion of hemolysins), iron-uptake systems (siderophores and iron mimetics), and post-iron-uptake systems (heme degradation).

### 4.1. Targeting Pre-Iron-Uptake Systems

#### 4.1.1. Quorum Sensing Inhibitors 

The production and secretion of hemolysins is part of a more generic mechanism, called quorum-sensing (QS), by which *S. aureus* undergoes a transformation from harmless to virulent for the host organism [118,206]. Hla and Hld are mainly positively regulated by the accessory gene regulator (*agr*) locus, part of a QS system [207]. The *agr* operon consists of four genes (*agrBDCA*) that encode for the respective AgrBDCA proteins. For a more detailed description of this QS system please refer to [208,209,210].

Administration of quorum-sensing inhibitors (QSI) or quorum-quenching agents (QQA) leads to inhibition of virulence factors expression, which makes bacteria less aggressive and more susceptible to natural immunity. Antivirulence agents are neither bactericidal nor bacteriostatic and in principle should be less susceptible to resistance. Many efforts have been done in that direction and, even though there is currently no antivirulence agent in clinical use, several studies show the usefulness of these compounds, alone or as adjuvants in conventional antibiotic therapy for methicillin-resistant *S. aureus* (MRSA) infections. Indeed, the combination of an antibiotic with an antivirulence agent constitutes a potential new mode of treatment that may alleviate the antibiotic resistance crisis. 

Ideally, inhibition of the *agr* operon and the toxin production can be achieved by targeting any component of the system. The most exploited strategies are reported hereafter.

The interaction between the cytoplasmic protein AgrA, acting as a transcription factor of *agr* operon, and the bacterial promoter has been extensively studied. The AgrA C-terminal (AgrA_C_) DNA-binding domain LytTR was identified as a druggable site, with the aim to inhibit the engagement with the bacterial DNA. In the same study, a screening of 500 fragment compounds led to the identification of three molecules able to inhibit AgrA DNA binding activity in the range of 10^−4^ and thus can be considered a good starting point for further drug development [211,212,213]. 

A series of biarylhydroxyketones was discovered in 2013 to inhibit in vitro MRSA-triggered erythrocyte hemolysis by 98% at a concentration of 1 µg/mL [214,215]. The survival benefit of the treatment with these compounds was also assessed in vitro and in vivo, alone or in combination with β-lactam antibiotics to which MRSA is resistant in monotherapy. Notably, even though monotherapy with QQA did not significantly reduce bacterial load compared to control mice, it did confer 100% survival and relatively good health status to animals inoculated with a lethal dose of MRSA, thus potentially representing a novel non-antibiotic therapy [216,217,218].

In 2014, Sully and colleagues [219] identified savirin (*S. aureus* virulence inhibitor) as an inhibitor of the *agr*-specific transcription regulation of the major virulence factors, such as Hla, with promising outcomes. Interestingly, the compound was also shown to not be subjected to tolerance or resistance mechanisms. The same group also investigated a series of nine polyhydroxyantraquinones, produced by *Penicillium restrictum*, able to bind the AgrA_C_ DNA binding region, among which Ω-hydroxyemodin (OHM) was found to reduce dermonecrosis in mouse model of MRSA skin infection [220,221]. The exploration of other bioactive natural products led to the identification of apicidin, a cyclic tetrapeptide fungal metabolite which, again, demonstrated suppression of *agr* activation through AgrA inhibition [222]. 

Finally, mimetics of the autoinducing peptides (AIPs), which are fundamental for assessing bacterial population density and interact with AgrC receptors [223,224], have been proposed as possible novel antimicrobials with extensive structure–activity analysis [225,226,227,228,229].

One concern in the clinical use of QSI is that their efficacy is related to the immune system integrity (bacterial clearance is host-mediated), meaning that in immunocompromised patients the only use of QSI could not be enough to overcome an infection. 

#### 4.1.2. Small Molecules as Hemolysin Inhibitors

Few hemolysin inhibitors have been reported till now, with mechanistic details mainly explained by means of in silico studies. According to their mechanism, hemolysin inhibitors may be divided into two groups: (i) hemolysin monomer binders, which hinder the oligomerization process, and (ii) direct blockers of the pore formed by the oligomerized Hla subunits. Natural compounds such as baicalin, cyrtominetin and oroxylin A [230,231,232,233], and peptides [234] are included in the first group, whereas the second group is mainly represented by beta-cyclodextrin derivatives [235,236] or complexes [237], heparins [238], and isatin-Schiff base Cu(II) complexes [239]. Overall, hemolysin inhibitors effectively prevent hemolysis in vitro, but without any anti-staphylococcal activity. To date, no lead compound among Hla inhibitors has been identified. 

#### 4.1.3. Iron Chelators

A number of iron-chelating agents, clinically useful in treating iron-overload conditions, have been evaluated for their ability to inhibit bacterial growth in multidrug-resistant bacteria, mainly by competing with bacterial siderophores for available iron.

DFO (Desferal^®^, Novartis), a hexadentate hydroxamate siderophore derived from *Streptomyces pilosus*, was one of the first compounds to be investigated as an inhibitor of the in vitro growth of various Staphylococci, including *S. aureus*. However, Staphylococci appeared to give different responses to DFO treatment, with the growth of many *S. aureus* strains being actually enhanced [240], due to the ability of these bacteria to capture the xenosiderophore complexed with iron and eventually use it as an iron source.

**Figure 3:**
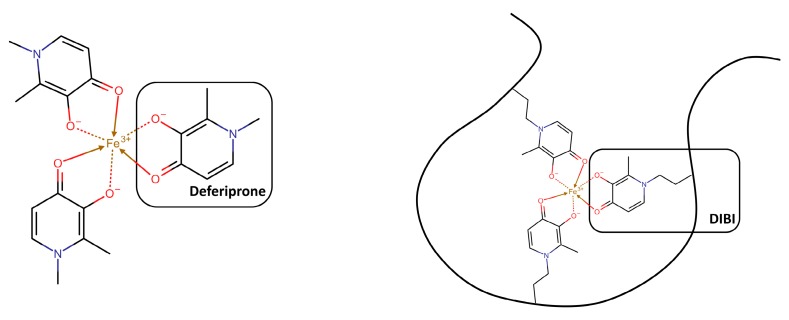
Schematic representation of Deferiprone (Ferriprox^®^) and DIBI in their iron-bound forms (adapted from [249]). The chemical structures were drawn with Chemicalize.

Deferiprone (Ferriprox^®^), a bidentate 3- hydroxypyridin-4(1H)-one (3,4-HOPO) chelator (Figure 3), is a more recent synthetic, FDA-approved, oral chelator for iron overload due to blood transfusions in thalassemia major patients. Even though at high concentration, with MICs typically >68 µg/mL (>0.5 mM), it was found to inhibit - or at least to not promote - the growth of Staphylococci including *S. aureus* [240,241]. Deferiprone showed a synergic effect when tested in combination with the heme analogue Ga protoporphyrin against *S. aureus* biofilms in in vitro and in vivo infection models [242,243]. It has been speculated that the low activity of deferiprone is due to the ability of bacteria to passively internalize it, as a result of its low molecular weight. The strategy followed to improve the antimicrobial activity of deferiprone was to either chemically link many 3,4-HOPO iron-binding moieties to give hexadentate dendritic chelators, or to incorporate them into a sufficiently large linear polymer, in order to make it unrecognizable and less accessible for bacterial internalization [244,245,246,247,248]. DIBI, a 3,4-HOPO derivatized water-soluble poly-vinylpyrrolidone co-polymer (Figure 3), displayed more than 70 times lower MIC values in vitro than the chemically related deferiprone. DIBI was also evaluated in vivo, where it exhibited a dose-dependent suppression of infection, as well as reduced wound inflammation and staphylococcal burden. When administered in combination with sub-MIC concentrations of the antibiotics gentamicin, ciprofloxacin, and vancomycin (conditions generally favorable for microbial re-growth/infection and positive selection of antibiotic-resistant survivors), the Fe-restricted growth resulting from the presence of DIBI enhanced the overall activities of the three antibiotics and promoted additional and prolonged bacterial killing [249].

The Deferiprone chemically related iron-chelating moiety 1-hydroxy-2(1H)-pyridinone (1,2-HOPO), linked to a triaza-macrocyclic backbone scaffold to give a hexadentate chelator, was also evaluated for its inhibitor activity against Gram-negative and Gram-positive bacteria, among which *S. aureus* [250].

Despite their unquestionable ability to chelate and thus eliminate the free iron as a source of bacterial nutriment, these compounds exhibit relevant MICs decrease only in combination with antibiotics. This could be attributed to the many redundant mechanisms that *S. aureus* possesses to supply iron deficiency, including the expression of the Isd system proteins.

### 4.2. Targeting the Iron-Uptake Systems

#### 4.2.1. Exploiting the Iron-Uptake System

##### Trojan Horses 

**Figure 4:**
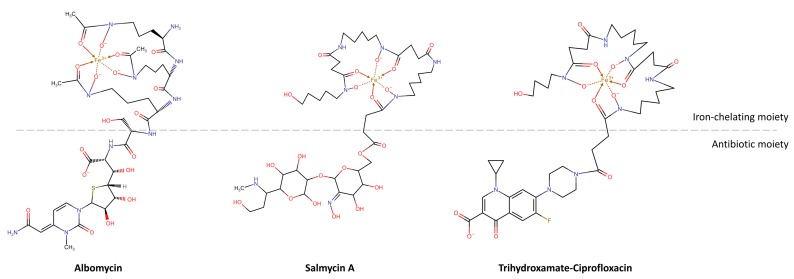
Schematic representation of the state-of-the-art Trojan horses (adapted from [261,262]). The chemical structures were drawn with Chemicalize.

Siderophores have been envisaged as “Trojan horses”, for their capacity of entering bacteria and deliver therapeutic agents conjugated to the siderophore molecule (Figure 4). Even if covalently conjugated with other molecules, for instance, antibiotics, siderophores are often recognized by their transporters, which pull them inside bacterial cells. Here the conjugated molecule can be released and exert its action [251,252,253]. 

This strategy has been first exploited by the same bacteria for delivering antibiotics in competing organisms able to internalize xenosiderophores. These conjugated compounds, constituted by a siderophore and a covalently bound antibiotic, were identified for the first time in 1947 in *Streptomyces* strains [254], before the discovery of siderophores. They were found to compete with the siderophore uptake system and first named sideramines or siderochromes. They were later referred to as sideromycins, and their structure was properly defined only in 1982 [255]. 

Albomycins, the first discovered sideromycins, present a ferrichrome-like trihydroxamate siderophore linked to a seryl-thioribosyl pyrimidine. They inhibit seryl-tRNA synthetase and have a broad-spectrum activity towards both Gram-positives and Gram-negatives, with MIC in the order of 5 ng/mL [251,256]. The FhuA and FhuD ferrichrome membrane transporters, capable of actively transport siderophores and siderophore derivatives through the bacterial cell, are responsible for this high potency. Once in bacteria, the antibiotic is released by a serine protease, which cleaves the amide bond linking the two moieties. While the antibiotic remains within the cell, the iron-free carrier is released.

Salmycins were later isolated from a *Streptomyces violaceus* strain [257]. They are formed by a linear trihydroxamate siderophore (danoxamine) belonging to the ferrioxamine family, a succinoyl linker and an aminoglycoside, which inhibits protein synthesis. Differently from albomycins, salmycins are selective towards Gram-positives, with MIC values in the order of a few ng/mL [251]. Again, the extremely high potency can be attributed to the specific transport mechanism that occurs by means of hydroxamate siderophore membrane transport proteins [258]. Unfortunately, the in vivo activity towards *S. aureus* is not so high and the compound has to be administered at shorter intervals with respect to other antibiotics as vancomycin and rifamycin, possibly because of the instability of the ester linkage [251]. Salmycins have been, instead, patented for treating iron-overload diseases [259,260]. Their applicability has been also supported by the incapability of mammalian siderophore-binding proteins, i.e., siderocalins, to bind ferrioxamine siderophores, and by the consequently high bioavailability.

Ferrimycins, finally, are constituted by a ferrioxamine B moiety, conjugated to an antibiotically active group, and specifically target Gram-positives [261].

As mentioned, despite the quite different nature of the antibiotic moiety, both albomycins and salmycins are transported by the same siderophore transport proteins. 

Sideromycins are equally expressed and potent in Gram-negatives, in which the outer membrane does not represent a permeability barrier, but actively facilitates their transport. For many antibiotics, in fact, the diffusion through the outer membrane is so poor that the MIC reaches toxic levels. Exploiting the siderophore transport system often leads to a 100-fold MIC reduction [263]. Indeed, the same seryl-thioribosyl pyrimidine moiety of albomycin, without a siderophore carrier, is 30,000-fold less potent against *S. aureus* and *E. coli*, because of permeability issues [251]. As well, the semi-synthetic CGP 4832 rifamycin derivative was found to be 200-fold more active than the original rifamycin in *E. coli* and *Salmonella* strains [264].

As for other antimicrobials, the same bacteria can become resistant to sideromycins, generally because of mutations at the level of the siderophore receptor or of the TonB transport complex. 

The capability of these conjugates to easily cross the bacterial membrane was soon exploited in antimicrobial research, and synthetic compounds mimicking natural sideromycins were designed using a “Trojan horse” strategy. These molecules contain three components, a natural or mimetic iron-chelating siderophore, a linker, and an antibiotic. With respect to canonical antibiotics, these siderophore–antibiotic conjugates generally present an improved selectivity, since each bacterium produces and uses different types of siderophores. A higher specificity goes in the direction of more responsible use of antibiotics, because of a reduced risk of antibiotic resistance. Other possible advantages are related to the enhanced antibacterial potency and the selectivity for pathogenic strains over non-pathogenic ones, accomplished by the transformation of Gram-positive antibiotics in Gram-negative antibiotics, which might render multiple drug-resistance bacteria more susceptible [265].

Thanks to the structural tolerance displayed by siderophore transporters, artificial siderophores, and conjugates quite different from the parent structures can be transported as the natural siderophores in the targeted bacterial cells. However, the best strategy for the development of a successful synthetic conjugate relies on the replication of natural siderophores [266,267]. Accordingly, catecholates and hydroxamates constitute the iron-chelating portion of the majority of the known siderophore–antibiotic conjugates, being recognized as natural or xenosiderophores by both Gram-negative and Gram-positive bacteria, respectively [99]. Regarding the linker, a fine balance of chemical reactivity is required, since it has to be sufficiently stable in the extracellular environment, but cleavable once in the cytoplasm [265]. Finally, β-lactam antibiotics have provided, up to now, the most successful conjugates targeting Gram-negative bacteria. However, conjugates containing fluoroquinolones have been also developed to cross the inner membrane and reach cytoplasmic targets of Gram-negative and, in particular, of Gram-positive bacteria [268]. 

Among the first to design semisynthetic siderophore–antibiotic conjugates, Zahner and coworkers joined a sulfonamide antibiotic to ferrioxamine B analogs, obtaining compounds with a limited spectrum of action against *S. aureus* [269]. Later, Ghosh and Miller combined catechol and mixed catechol-hydroxamate ligands with the Gram-positive antibiotic vancomycin, to synthesize conjugates that showed a reduced activity with respect to the antibiotic alone [270]. Differently, a hydroxamate-5-fluorocytosine conjugate, properly designed to undergo ester-mediated cleavage, demonstrated enhanced activity towards *Staphylococcus* and *Enterococcus* spp., when compared to the 5-fluorocytosine active metabolite alone [271].

Inspired by natural salmycins, Wencwicz and colleagues designed a series of linear hydroxamate siderophore-fluoroquinolone conjugates to target *S. aureus* [262]. The authors started from the salmycin siderophore portion containing a succinoyl linker and synthesized six derivatives with an increasing number of hydroxamate groups (from one to three), conjugated to the broad-spectrum β-lactam carbacephalosporin Lorabid, having a periplasmic biological target, and to the broad-spectrum fluoroquinolone ciprofloxacin, targeting the cytoplasmic DNA gyrase. In general, the β-lactam derivatives demonstrated a narrower spectrum of action and a reduced potency against the ESKAPE bacteria group (*Enterococcus faecium*, *S. aureus*, *Klebsiella pneumoniae*, *Acinetobacter baumannii*, *P. aeruginosa*, and *Enterobacter* species). Moreover, the mono- and bis-hydroxamate fluoroquinolone conjugates also had a reduced activity spectrum and a reduced potency with respect to ciprofloxacin, while the trihydroxamate derivative showed selectivity towards Gram-positive *S. aureus*, maintaining a similar potency in terms of MIC (1 mM with respect to 0.5 mM). The compound, thus, is one of the very few synthetic siderophore–antibiotic conjugates capable of maintaining the activity of the original antibiotic. The narrower spectrum of action must be attributed to the trihydroxamate siderophore, also known as desferridanoxamine, better recognized, with respect to the mono- and bis derivatives, by FhuD1 and FhuD2, the promiscuous membrane-anchored hydroxamate siderophore binding proteins of *S. aureus*. Gram-negatives have more selective siderophore binding outer-membrane proteins, possibly unable to recognize these derivatives. The lack of activity of β-lactam conjugates could be attributed to the location of the biological target. As the compounds are transferred by active transport into the cytoplasm, a target as PBPs located in the plasma membrane is not the ideal one. Thus, when targeting Gram-positives with siderophore–antibiotics conjugates, cytoplasmic targets should be preferred. The capability of the trihydroxamate-ciprofloxacin derivative to reach the target by means of active transport was also demonstrated by the dependence of the antibacterial activity on iron concentration and the presence of siderophores competing for the same transport mechanism. As stated by the authors, two main advantages can be associated with the development and use of trihydroxamate-ciprofloxacin derivatives. First, the selectivity of the compounds towards *S. aureus* makes them ideal candidates for targeted antimicrobial chemotherapy, which limits the exposure of other bacteria to ciprofloxacin and, thus, is less prone to generate multidrug resistance. Moreover, the compound intrinsic toxicity towards mammalian cells is reduced because siderophore transporters are absent in eukaryotic cells. On the other side, the limits associated with “Trojan horse” compounds could be represented by a reduced efficacy in treating intracellular infections because of the incapability of passing eukaryotic cell membrane. Also, the use of a very selective treatment implies an early detection of the infective agent, with respect to the use of broad-spectrum antibiotics.

In the same period, Milner and coworkers attempted to join the fluoroquinolones ciprofloxacin and norfloxacin to the same siderophore produced by *S. aureus*, that is SA [272]. With respect to other siderophores, staphyloferrin has a higher iron affinity because of its hexadentate nature, is highly hydrophilic and is an efficient chelator in mildly acidic environments. Unfortunately, when tested on a panel of 19 reference and clinical isolates, the compounds showed no activity towards ciprofloxacin- and norfloxacin-resistant strains, and a very reduced potency with respect to the original fluoroquinolones. 

When designing a new siderophore-conjugate, Ji and Miller chose DFO as siderophore, ciprofloxacin as antibiotic and a trimethyl-lock based linker, specifically designed to promote the antibiotic release by means of esterase or phosphate-mediated hydrolysis [273]. The trimethyl lock is an o-hydroxycinnamic acid derivative functionalized with three methyl groups, which, by steric hindrance, induce lactonization and consequent antibiotic release, if triggered by potential esterases or phosphatases. Ciprofloxacin was chosen again as antibiotic because of the wide spectrum of action and the intracellular target, while DFO was selected as a well-characterized trihydroxamate siderophore produced by a number of bacterial species. Two different conjugates were prepared, plus a control compound having a stable succinyl linker. When tested for their capability to inhibit bacterial growth in agar well diffusion tests, the two conjugates demonstrated moderate activity against Gram-negative and Gram-positive strains, including *S. aureus*. The control compound was, instead, not active, suggesting it was no more recognized by the membrane siderophore receptors. MIC analyses confirmed a moderate antibacterial activity against all the strains with the exception of *E. faecium*. However, in general, the authors observed a decreased activity with respect to the parent antibiotic and attributed it to the compounds being poor substrates for esterases. To overcome the risk that the conjugates could be cleaved by extracellular hydrolases, the same authors proposed similar conjugates bearing a linker cleavable by ferric reductases, instead of esterases or phosphatases [274]. Upon transfer of the compound within the bacterial cell, the hydride donors that reduce Fe(III) to Fe(II) should also reduce the quinone linker, generating a transient hydroquinone, rapidly converted into lactone because of the “trimethyl lock”. The siderophores were DFO and a mixed biscatecholate-monohydroxamate ligand designed to exploit multiple siderophore recognition processes. The conjugated quinolone was ciprofloxacin. The conjugates were found, again, less active with respect to the parent drug in both Gram-positives and Gram-negatives, possibly because of a more difficult recognition by siderophore transporters, and a less efficient linker activation within the bacteria. Again, the authors supported the use of reduction-triggered linkers with respect to more stable ones. 

The failure of “Trojan horses” strategies could be attributed to the same specific uptake systems that make these conjugates particularly active. Indeed, highly modified conjugates could result more difficult to be transported than native compounds. The presence in bacterial cells of several redundant iron-uptake systems, able to switch off when needed without affecting the cell survival, represents an additional failure reason, as well as the necessity to further release from the conjugate the antibiotic, by means of peptidase- or esterase-mediated cleavage [261]. Fortunately, the chemical space available for the design of new and more potent conjugates is still very wide. More than 500 different siderophores have been reported in the literature, and antibiotics other than β-lactams or DNA-gyrase inhibitors could be attached to them. 

##### Gallium-Derivatives

Gallium-based molecules have been also classified as “Trojan Horses” for their capacity of being recognized by the iron transport system. Good antibacterial activities have been shown by simple gallium salts as nitrate (GaN) or maltolate (GaM) [275,276,277,278,279], and by more complex gallium siderophores as gallium citrate or by gallium-containing porphyrins [280]. 

Fe(III) and Ga(III) have a significant chemical similarity, concerning ionic radius, electronegativity, ligand affinities, and coordination geometry [281]. Accordingly, Ga(III) can be exchanged for Fe(III) in siderophores, blocking bacterial iron acquisition [282]; it can be taken up as a gallium siderophore exploiting the iron transport system, perturb the iron acquisition system acting on siderophore transcriptional regulator, or interfere with the biofilm formation [283,284].

Despite the high similarity, however, Ga(III) cannot be reduced in physiological conditions; thus, any biological process involving an iron redox reaction, as electron transport or oxidative stress defense, can be disrupted by the iron-gallium substitution [281,285]. Also, gallium-derived compounds display quite different pharmacokinetics. For instance, the elimination of Ga-citrate is slower than that of Fe-citrate and the volume of distribution is six times higher. Interestingly, these compounds are not expected to be affected by the standard drug resistance mechanisms, as decreased uptake, restricted bacteria permeability or efflux pumps expression, thus representing interesting strategies to fight antimicrobial resistance. 

Gallium nitrate has been extensively tested towards both Gram-negatives as *P. aeruginosa* and Gram-positives as *S. aureus*. It is well distributed in the body thanks to its binding and transport by transferrin, and easily taken up by bacteria through the iron transport system. Moreover, it has been proved capable of disrupting and preventing the formation of biofilms [283]. More complex systems include gallium citrate, tartrate [281], pyridones [286], acinetoferrin analogs [287], and non-iron metalloporphyrins (MPs) [288]. The complex formed by gallium and SA, unfortunately, resulted inactive because internalized with difficulty by *S. aureus*. 

**Figure 5:**
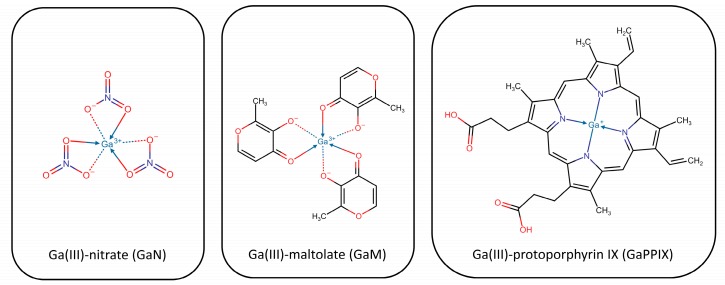
Representation of Ga-derivatives. The chemical structures were drawn with Chemicalize.

The most promising MPs were first described by Stojiljkovic and colleagues in 1999 [288]. Once taken up by bacteria heme uptake systems, MPs target the enzymes of metabolic pathways, which become unable to properly reduce oxygen and induce the production of reactive oxygen species (ROS). MPs demonstrated to reduce MICs towards different bacteria, including multidrug-resistant ones. In particular, non-iron protoporphyrin IX including gallium, indium, and manganese was more active towards *Yersinia enterocolitica* and *Mycobacterium smegmatis*, while that including zinc and ruthenium demonstrated prominent activity against *S. aureus* [289]. The most promising activity was shown by Ga-PPIX (Figure 5), probably because of its high structural homology with the iron ion and its poor toxicity. 

Richter and coworkers recently demonstrated that the combined treatment with Ga-PPIX and the iron chelator deferiprone significantly reduces the growth of methicillin-susceptible *S. aureus* (MSSA) and MRSA [290], and small *S. aureus* colony variants [243]. Apart from the positive observed results, it should be reminded that Ga-PPIX-mediated treatments could be cytotoxic because of the possible interference with host iron metabolism. Researchers observed a loss of cell viability and an increase in the production of lactate dehydrogenase in cell lines exposed to high Ga-PPIX concentrations [288,290,291]. It is likely that the Ga-PPIX concentration required to inhibit biofilm formation is lower than that responsible for cytotoxicity [243], which suggests that a proper balance between antimicrobial activity and cytotoxicity could be found [292].

Along with Ga-PPIX, also Ga-deuteroporphyrins, Ga-mesoporphyrins, Ga-hematoporphyrins, Ga-octaethylporphyrins, and Ga-porphyrins have provided interesting activity towards MRSA [289]. Bacteria not targeted by MPs include strict fermenting anaerobes, which do not use heme uptake systems or cytochromes.

More recently, the antimicrobial activity of Ga-containing compounds was confirmed by Hijazi and coworkers towards the ESKAPE pathogens, including reference strains and clinical multidrug-resistant (MDR) ones [293]. The authors compared the activity of three generations of Ga(III) formulations, i.e., GaN, GaM, and Ga-PPIX, in culture media having different iron content. The compounds were active against *S. aureus* and *A. baumannii* strains. In particular, GaN and GaM showed a bacteriostatic effect, while Ga-PPIX had a bactericidal activity on some strains. Interestingly, the latter demonstrated to be susceptible to the number of bacterial heme uptake systems and the presence of serum albumin, able to bind a variety of ligands, including heme, and to inhibit their activity. They also confirmed the susceptibility of all derivatives to the iron concentration (enhanced by iron deprivation) [285], and of Ga-PPIX to the heme concentration. Indeed, the addition of a 5 mM concentration of heme was enough to completely abrogate Ga-PPIX activity in *S. aureus* strains. Overall, being active even in the presence of serum albumin, GaN and GaM derivatives were the most effective in in vivo mimicking conditions. It is interesting to note that Ga(III) derivatives already proved to be effective in infections caused by Gram-negatives [276,294,295].

Apart from Ga-PPIX, Mn-PPIX and Zn-PPIX also exhibit antimicrobial activity against *S. aureus* [296]. As already mentioned, Ga-PPIX - but also Zn-PPIX - exert their antimicrobial activity by inhibiting aerobic respiration through the substitution of heme in cytochromes (a property also shared by non-toxic non-iron MPs), but also by causing oxidative stress. Interestingly, toxic non-iron MPs induce the expression of *S. aureus* heme detoxification system. Upon exposure to heme toxic levels, in fact, the heme sensor system HssRS is activated and the heme-regulated transporter HrtAB is expressed [176]. However, HrtAB is unable to detoxify many toxic non-iron MPs. Because of the massive upregulation, its expression becomes detrimental and increases the bactericidal activity of these compounds [296].

Photoactive porphyrins have been also used as photosensitizers for the photodynamic inactivation of microorganisms, based on the generation of singlet oxygen species and free radicals, upon the activation of the porphyrins by visible light [297]. A cationic tetra-N-pyridyl and a peripheral [Pt(bpy)Cl]^+^ substituted porphyrin were evaluated against *S. aureus* and *E. coli* strains in dark and light conditions, showing a lower activity in dark conditions. This supported the photodynamic mode of action of the compounds [298]. Their efficacy is associated with the interaction they can form with membrane negative charges that promote porphyrin permeation and accumulation in membranes, where reactive oxygen species can be generated upon light irradiation.

Overall, Ga-derivatives might represent a powerful resource for the development of new antimicrobials, to be administered alone or in combination with conventional antibiotics, as recently demonstrated by Richter and coworkers [242].

#### 4.2.2. Inhibiting the Iron-Uptake System

In principle, small molecules inhibiting the iron-uptake system may target either siderophore receptors or hemophores. SA and SB receptors (lipoproteins HtsA and SirA, respectively) bind their ligands with nanomolar affinity, so that competitive inhibition is precluded. As for xenosiderophores receptors, up to now, no work has been carried out for investigating whether SstD, or FhuD1 and FhuD2, are druggable with small molecules. Nonetheless, staphyloferrins and xenosiderophores receptors have been addressed as promising antigens for staphylococcal vaccines [109] (see Section 5).

The novelty and potential of the recently characterized IsdA-I hemophores have been arousing interest lately: at the time of writing, hemophores have not been addressed yet as biological targets for small molecules, although the literature extensively reports their role as antigens in vaccine formulations (see Section 5) and many authors have suggested their potential exploitation [147,299,300,301]. Finally, sortases represent another potential target for therapeutic intervention since their inhibition is expected to lead to the mistargeting of cell wall-associated proteins. While inhibitors of SrtA should inhibit the correct localization of many virulence factors in the cell wall (see [302] for a perspective on this topic), inhibitors of SrtB are expected to more specifically affect iron acquisition through IsdC. Furthermore, while SrtA is expressed by all Gram-positive bacteria, SrtB is more specific to *S. aureus*, *Bacilli,* and *Listeria*. However, deletion of SrtB has only mild effects on *S. aureus* virulence (see Table 2) and a limited number of works have been reporting positive results on inhibitors development [303,304].

##### Inhibitors of Siderophore Biosynthesis

The siderophore machinery, being one of the main and most effective systems for iron acquisition, has been attacked from several fronts with the aim of developing new antimicrobials. 

The biosynthesis of staphyloferrin B has been targeted by Tripathi and co-workers [305]. In particular, the authors targeted the type A NIS synthetase SbnE, which catalyzes the condensation of citric acid with L-2,3-diamino propionic acid [84]. By screening a library of marine microbial-derived natural product extracts, they identified two new antibiotics named baulamycins A and B (BmcA and BmcB, respectively). The two molecules demonstrated to act as reversible competitive inhibitors towards SbnE of *S. aureus* but also towards AsbA of *B. anthracis* with IC_50_ in the micromolar range. When tested in liquid bacterial cultures they were able to inhibit the growth of both Gram-positive and Gram-negative bacteria, thus proving the potential of siderophore virulence factor inhibitors as antibiotics. Being type A NIS synthetases a class of enzymes common to many pathogenic bacteria, they represent suitable targets for the development of broad-spectrum agents. The strategy of inhibiting siderophore biosynthesis has been also proven successful in previous studies targeting *Mycobacterium tuberculosis* [306,307,308].

### 4.3. Targeting the Post-Iron-Uptake Metabolism 

#### Heme Oxygenase Inhibitors

In a recent article, Conger et al. [301] reported the differences between IsdG and IsdI biological roles, with the former being overexpressed in iron-restricted environment, the latter being constitutive and limiting iron toxicity, and consequently addressing IsdG as the preferred antibiotic target. Furthermore, the authors reported low nanomolar dissociation constants for heme-IsdG complexes, hence excluding the design of competitive inhibitors. Research tracks for IsdG uncompetitive or allosteric inhibitors, however, remain open. In particular, even if lacking drug-likeness and suffering from consistent off-target effects, uncompetitive inhibitors such as cyanides and azides have been investigated [309], while no allosteric inhibitor has been reported yet. Lastly, Conger and colleagues suggested continuing the exploration of IsdG and IsdI mechanism of action to better design specific inhibitors [301].

## 5. Biopharmaceutical Approaches Targeting the Iron System in *S. aureus*

Biopharmaceuticals are defined as pharmaceuticals obtained by biotechnological processes and include, inter alia, recombinant proteins, gene therapy, allergenics, living cells, blood components, antibodies, and vaccines. Among other benefits with respect to small molecules, biopharmaceuticals are highly specific and could not only treat but also prevent infectious diseases. Hence, as antibiotic-resistant strains are emerging at an alarming rate, *S. aureus* has become an enticing target for biopharmaceuticals. Among these, vaccines may lower the morbidity and mortality rate of staphylococcal infections, protecting high-risk patients undergoing periodic procedures such as hemodialysis or invasive surgeries and, ultimately, alleviating the clinical burden. There have been multiple attempts to create a vaccine against antigens belonging to the staphylococcal iron metabolism but, up to now, all of them failed to reach the market [310]. Anti-toxin antibodies are another beaten research track for reducing or treating staphylococcal infections, neutralizing widespread antigens in clinically relevant *S. aureus* strains. Furthermore, in this case, to date, no approved therapy is still available. In this paragraph, the two most relevant macro areas of biopharmaceutical research against *S. aureus* are addressed, i.e., the iron/heme uptake system and hemolysins. 

### 5.1. Biologics Targeting the Staphylococcal Iron/Heme Uptake Systems

An early attempt of a vaccine against components of the iron metabolism was reported in 2006 [311] and was directed towards the cell-wall anchored hemophore IsdB, whose antigenic potential was discovered in a large in vitro screening of human sera against a library of *S. aureus* epitopes [312]. Furthermore, IsdB is an extremely appealing target as it is conserved among different clinically relevant strains and highly expressed in vivo, both in sensitive and resistant bacteria [311]. Hence, remarkable efforts were made for developing a prophylactic monovalent vaccine against IsdB (V710, by Merck Sharp & Dohme Corp) between 2006 and 2011, to prevent *S. aureus* infections. In the preclinical phase, inoculation of purified IsdB had a protective effect on murine models challenged with several lethal and sub-lethal doses of *S. aureus*, while rhesus macaque species responded to immunogenic assays with long-lasting anti-IsdB titers [311]. Unfortunately, even if phase I (NCT00303069, NCT01324440, NCT00822757, NCT00735839) and phase II (NCT00572910) clinical trials successfully proved the safety, immunogenicity, and non-toxicity of V710 [313,314,315], the anti-IsdB vaccine pipeline was discontinued in phase II/III. In fact, a double-blind placebo-controlled worldwide trial (NCT00518687) highlighted not only that administration of V710 did not exert a protective effect against *S. aureus*, but also that V710-induced antibody response could be linked to a five-fold higher mortality by post-surgery staphylococcal infections, compared to the placebo group [316].

The paradoxical results of V710 phase III trial were a matter of speculation in articles and retrospective studies [317,318,319]. McNeely et al. [318] pointed out the positive correlation between low serum levels of interleukins IL2/IL17a before and after vaccination and increased mortality rates by post-operatory *S. aureus* infections. In the same work, the authors hypothesized that V710 may trigger an original antigenic sin mechanism, hence causing a suboptimal or even ineffective response when the organisms are challenged with *S. aureus* natural infections. Other remarkable limitations possibly explaining the detrimental outcome of V710 vaccine include overrating the humoral immunity for preventing *S. aureus* infections while neglecting the importance of cellular-mediated immunity [316,320], and discoverable immune idiosyncrasies, which may heavily impact the efficacy of staphylococcal vaccines [318]. At the time of writing, the exact physio-pathological mechanism underlying the increased mortality caused by V710 in *S. aureus* infected patients is still missing, and this lack of knowledge has been linked to lurking variables not documented in the original study, such as the site and the severity of the post-operatory infections [318]. Importantly, for overcoming the limitations encountered by V710 and for designing more effective next-generation vaccines against *S. aureus*, recent literature [320] suggested refinements to be adopted for future research, such as a multi-antigen approach, preclinical studies involving infections models other than murine, or adopting different strategies depending on the site of infection. Parallel to the development of V710, research evaluating the addition of IsdA in multi-antigen vaccines has been carried out in rodent models, but without any further follow-up in clinical trials to date [151,321,322]. 

Extra work has been carried out to characterize the human humoral response to *S. aureus* and IsdB in particular [323,324]. In 2016, Yeung et al. [323] detailed that the adaptive immune response can encode antibodies with motives inherently able to recognize NEAT1 and NEAT2 domains in IsdB. Antibodies encoded by germline genes IGHV4-39 and IGHV1-69 have been reported to have an almost identical binding mode on IsdB NEAT1 and NEAT2 domains in X-ray crystal structures deposited in Protein Data Bank (PDB codes 5d1q, 5d1x, 5d1z [323]). Similarly, in a more recent work, Bennet and coworkers [324] described at least three binding sites for IGHV1-69-encoded monoclonal antibodies (mAb) on IsdB NEAT2. In particular, mAb STAU-281 demonstrated a promising protective effect on liver, heart, and kidneys when tested on mice. The authors further reported the binding mode of three mAbs on NEAT2 domain, where loops CDR-H2 and CDR-H3 are mainly involved in the interaction with the heme pocket.

Recent studies [66,110,325] identified the widespread and conserved staphylococcal iron-hydroxamate-binding lipoprotein FhuD2 as another promising vaccine-target candidate, inducing a protective immune response in murine abscess and sepsis models. Learning the lesson from the outcomes of the monovalent V710, the authors suggested using FhuD2 antigen as a part of multicomponent vaccines and, accordingly, in late works FhuD2 has been included in multi-antigen formulations [326,327].

### 5.2. Biologics Targeting Staphylococcal Hemolysins 

Biologic products against hemolysins make up the second substantial part of biopharmaceuticals against the staphylococcal iron system and consist of either immunotherapeutics or vaccine candidates. Significantly, in vivo tests demonstrated avirulence of staphylococcal strains deficient in hemolysin production [123].

The literature focuses on neutralizing antibodies against hemolysins (Hla in particular) as a possible platform for the development of active or passive immunization therapies against *S. aureus* infections [328,329,330,331]. Hua et al. [329] demonstrated that the administration of anti-Hla antibodies not only reduces murine abscess models, but also prevents and treats lethal *S. aureus*-induced pneumonia in mice. Furthermore, anti-Hla mAbs have been successfully combined with last-generation antibiotics, such as vancomycin and linezolid, enhancing or synergizing their activity [329,331]. Experimentation on ASN100, a monoclonal antibody targeting alpha-hemolysin and other cytokines, was terminated after a phase II trial (NCT02940626), due to its ineffectiveness in preventing pneumonia [332]. IBT-V02 is a preclinical heptavalent vaccine candidate in the Carb-X pipeline [333].

Currently, there are two vaccine candidates against Hla as promising adjunct treatments for *S. aureus*-induced nosocomial pneumonia, namely suvratoxumab (MEDI4893, an mAb sponsored by MedImmune LLC - now AstraZeneca), that proved to be safe and effective in a phase II study (NCT02296320) [334], and tosatoxumab (commercial name Salvecin™, previously AR-301 or KBSA301, by Aridis Pharmaceuticals), a human mAb that successfully completed phase I/II clinical trials (NCT01589185), currently under phase III evaluation (NCT03816956) [335].

The interaction between Hla and a human-derived antigen-binding fragment (Fab) has been characterized by Foletti et al. [331], who reported the crystal structure of Hla bound to a hinge lined by CDR-H1, CDR-H2 and CDR-H3 loops of the antibody heavy chain (PDB code 4idj). Rouha et al. described a multi-specific human mAb able to bind Hla and other four cytolysins [336].

Overall, other hemolysins aroused far less interest. These include a neutralizing antibody targeting Hlb reported by Pooja and coworkers [337] and a bivalent antibody binding HlgC described by Laventie et al. [338].

## 6. Conclusions

Targeting the iron-acquisition systems shows promise in the discovery of new anti-staphylococcal agents since the approach should in principle allow to decrease the chance for resistance development and selectively hit pathogenic bacteria without disturbing host microbiota or promoting the insurgence of opportunistic infections by *Clostridium difficile*. The main issue affecting the development of effective new antimicrobials is related to the presence of several redundant iron-uptake systems that can switch on and off according to external stimuli, without affecting bacteria survival. Targeting the pre-iron-uptake system through QS inhibitors could represent a valuable strategy, in particular, if antivirulence agents are administered in combination with antibiotics. Still, QS inhibitor activity is strictly related to the human immune system integrity. Hemolysin inhibitors, even if preventing hemolysis, do not have anti-staphylococcal activity and iron chelators are, again, effective only in combination with antibiotics, since *S. aureus* can acquire iron in different ways. The iron uptake system has been widely investigated as a possible target, mainly through the design of Trojan horses, originally developed by bacteria to counteract competing organisms. These molecules could be ideal candidates for their high selectivity and reduced toxicity. Even if highly modified conjugates have resulted difficult to be transported, the available chemical space is large and more effective combinations can be evaluated in the future. Similarly, also Gallium derivatives can be recognized by the iron transport system and have proved their antimicrobial activity when administered alone or in combination with conventional antibiotics. Siderophores and free iron uptake are not the only targetable systems. Indeed, IsdB and IsdH hemophores represent another essential way of iron acquisition and, accordingly, a promising and yet unexplored resource for the design of new antimicrobials affecting heme scavenging. In the post-iron-uptake metabolism, heme oxygenases, in particular IsdG, offer interesting perspectives too. Above all, biopharmaceuticals represent, today, the most advanced and concrete possibility. Apart from the yet unexplained failure of V710, the monovalent vaccine against IsdB, two vaccine candidates, suvratoxumab and tosatoxumab, targeting hemolysin H1a, have proved their efficacy and safety in phase II clinical trials, and represent the most promising perspective in the fight against *S. aureus*. Finally, the development of new in vitro/in vivo models to test the efficacy of molecules in the early stages of development will be of great value to allow the identification and the fast progression in the discovery pipeline of the more promising candidates.

## Figures and Tables

**Table 1:** Protein Data Bank (PDB) accession codes of proteins involved in iron acquisition by *Staphylococcus aureus.*

PDB Codes	Protein	Reference
3lhs, 3li2	HtsA	[57]
3eiw, 3eix	HtsA	[58]
5d84, 5d85	SbnA	[59]
4m54, 4mp3, 4mp6, 4mp8, 4mpd	SbnB	[60]
4tv5	SbnG	[61]
5uje	SbnI	[62]
6knh and 6kni	SbnH	[63]
3mwf, 3mwg	SirA	[64]
4fna, 4fil	FhuD2	[65]
4b8y	FhuD2	[66]
5twb, 5twc	IruO	[67]
7ahl	Hla	[68]
3b07	Hlg	[69]
4q7g	LukD	[70]
3roh	LukE	[70]
3i41	β-toxin	[71]
2kam	δ-toxin	[72]
1t2p, 1t2w	SrtA	[73]
1ng5	SrtB	[74]
4lfd	SrtB	[75]
4xs0	IsdH-Hb	[76]
5vmm	IsdB-Hb	[77]
6tb2	IsdH-Hb-Hp	[78]
2ite, 2itf	IsdA	[79]
2o6p	IsdC	[80]
2q8q	IsdE	[81]
1xbw	IsdG	[82]
3lgn	IsdI	[83]

**Table 2:** *S. aureus* deletion mutants in animal models of infection.

System	Gene Cluster	Regulation	Strain	Deletion	Gene Contribution to Virulence in Vivo	Mouse Model of Infection	Evaluated District of Infection	Reference
*Heme acquisition*								
Sortases	*srtA*	Constitutive	Newman	*ΔsrtA*	Yes	C57BL/6, Swiss-Webster (intravenous)	Kidney	[183]
	*isdC-FsrtBisdG*	Fur	Newman	*ΔsrtA*	Yes	NMRI (intra-articular)	Joints, kidney, blood	[184]
			Newman, USA300	*ΔsrtA*	Yes	BALB/c (intravenous)	Kidney	[150]
			Newman D2C	*ΔsrtA*	Yes	BALB/c (mammary injection)	Mammary glands	[185]
			Newman	*ΔsrtA, ΔsrtA-srtB*	Yes	CD-1 (intraperitoneal),	Systemic, joints,	[186]
						NMRI (intravenous), C3H/HeJ (bladder), Sprague-Dawley rats (intravenous)	kidney, heart	
			Newman	*ΔsrtA, ΔsrtA-srtB*	Yes	NMRI (intravenous)	Joints, kidney	[187]
			Newman	*ΔsrtB*	Mild	Swiss-Webster (intravenous)	Kidney	[142]
			Newman	*ΔsrtB*	Mild	NMRI (intravenous)	Joints, kidney	[187]
			Newman	*ΔsrtB*	Mild	CD-1 (intraperitoneal), NMRI (intravenous), C3H/HeJ (bladder), Sprague-Dawley rats (intravenous)	Systemic, joints, kidney, heart	[186]
Isd	*isdA*	Fur	Newman	*ΔisdBH, ΔhtsA-isdE*	No	BALB/c (intranasal)	Lung	[188]
	*isdB*	Fur	Newman	*ΔhtsA-isdE*	Yes	BALB/c (retro-orbital)	Lung, heart, kidney	[188]
	*isdC-FsrtBisdG*	Fur	Newman	*ΔisdB, ΔisdA*	Yes	BALB/c (intravenous)	Kidney	[150]
	*orfXisdI*	Fur	Newman	*ΔisdC*	Mild	BALB/c (intravenous)	Kidney	[150]
	*isdH*	Fur	Newman	*ΔisdH*	No	BALB/c (intravenous)	Kidney	[150]
			Newman	*ΔisdB*	Yes	C57BL/6J (retro-orbital)	Heart	[149]
			Newman	*ΔisdB*	No	C57BL/6J (retro-orbital)	Liver	[149]
			Newman	*ΔisdA, ΔisdB, ΔisdC*	Yes	BALB/c (retro-orbital)	Kidney	[151]
			Newman	*ΔisdH*	No	BALB/c (retro-orbital)	Kidney	[151]
			Newman	*ΔisdG, ΔisdI,*	Yes	BALB/c (retro-orbital)	Heart	[159]
			Newman	*ΔisdG-I*	No	BALB/c (retro-orbital)	Kidney	[159]
			Newman	*ΔisdI*	Yes	BALB/c (retro-orbital)	Kidney	[159]
			Newman	*ΔisdG, ΔisdG-I*	No	BALB/c (retro-orbital)	Liver	[159]
			Newman	*ΔisdG, ΔisdI,*	Mild	BALB/c (retro-orbital)	Kidney, spleen	[56]
			Newman	*ΔisdG-I*	Yes	BALB/c (retro-orbital)	Kidney, spleen	[56]
			8325-4	*ΔisdH*	Yes	NMRI (intravenous)	Blood	[165]
				*ΔisdB, ΔisdB-isdH*				
				*ΔisdH*				
Hss	*hssRS*	Constitutive, activated by heme	Newman	*ΔhssR*	Mutation increase virulence	BALB/c (retro-orbital)	Liver	[175]
			Newman	*ΔhssR*	No	BALB/c (retro-orbital)	Spleen, kidney	[175]
Hrt	*hrtAB*	HssRS	Newman	*ΔhrtA*	Mutation increase virulence	BALB/c (retro-orbital)	Liver	[175]
			Newman	*ΔhrtA*	No	BALB/c (retro-orbital)	Spleen, kidney	[175]
*Endogenous siderophores*								
Staphyloferrin A	*sfaABC*	Fur	Newman	*Δsfa*	Yes	BALB/c (subcutaneous)	Skin	[88]
	*sfaD*	Fur	Newman	*Δsfa-sbn*	Yes	BALB/c (subcutaneous)	Skin	[88]
	*htsABC*	Fur						
			MW2	*ΔsfaA*	Yes	Swiss-Webster (intravenous)	Kidney	[89]
			Newman	*ΔhtsA-isdE*	No	BALB/c (intranasal)	Lung	[188]
			Newman	*ΔhtsA-isdE*	Yes	BALB/c (retro-orbital)	Lung, heart, kidney	[188]
			Newman	*ΔhtsB, ΔhtsC*	Yes	BALB/c (intravenous)	Kidney, liver	[53]
Staphyloferrin B	*sbnA-I*	Fur, SbnI	Newman	*ΔsbnE*	Yes	Swiss-Webster (intravenous)	Kidney	[90]
	*sirABC*	Fur	Newman	*Δsbn*	No	BALB/c (subcutaneous)	Skin	[99]
			MW2	*ΔsbnD*	Yes	Swiss-Webster (intravenous)	Kidney	[89]
			Newman	*ΔsbnG*	No	BALB/c (intravenous)	Heart, kidney, liver	[87]
			Newman	*ΔsbnG-citZ*	Yes	BALB/c (intravenous)	Heart, kidney, liver	[87]
			Newman	*Δsfa-sbn, Δhts-sir, Δsfa-sbn-sst,*	Yes	BALB/c (intravenous)	Heart, kidney, liver	[109]
				*Δhts-sir-sst*				
*Xenosiderophores*								
Hydroxamate	*fhuBGC*	Fur	Newman	*ΔfhuD2*	Yes	CD1 (intravenous)	Kidney, blood	[110]
	*fhuD1*	Fur	Newman	*ΔfhuBGC*	Yes	Swiss-Webster (intravenous)	Kidney	[189]
	*fhuD2*	Fur						
Catecholate	*sstABCD*	Fur	Newman	*Δsst*	Yes	BALB/c (intravenous)	Heart	[109]
*Inorganic iron acquisition*								
Fep	*tatAC*	Constitutive	RN1HG	*ΔtatAC, Δtat-fep*	Yes	BALB/c (intravenous)	Kidney	[170]
	*fepABC*	Fur						
